# Microstructure and Electrochemical Behavior of a 3D-Printed Ti-6Al-4V Alloy

**DOI:** 10.3390/ma15134473

**Published:** 2022-06-24

**Authors:** Zhijun Yu, Zhuo Chen, Dongdong Qu, Shoujiang Qu, Hao Wang, Fu Zhao, Chaoqun Zhang, Aihan Feng, Daolun Chen

**Affiliations:** 1 School of Materials Science and Engineering, Tongji University, Shanghai 201804, China; yuzhijun1210@163.com (Z.Y.); qushoujiang@tongji.edu.cn (S.Q.); aihanfeng@tongji.edu.cn (A.F.); 2 Aerospace Hiwing (Harbin) Titanium Industrial Co., Ltd., Harbin High Tech Zone, Harbin 150028, China; cccccczhuo@163.com; 3 School of Mechanical and Mining Engineering, The University of Queensland, Brisbane 4072, Australia; d.qu1@uq.edu.au; 4 Interdisciplinary Center for Additive Manufacturing, School of Materials and Chemistry, University of Shanghai for Science and Technology, Shanghai 200093, China; 5 Academy for Advanced Interdisciplinary Studies, Southern University of Science and Technology, Shenzhen 518055, China; 6 School of Materials, Sun Yat-sen University, Guangzhou 510275, China; acezcq@gmail.com; 7 Innovation Group of Marine Engineering Materials and Corrosion Control, Southern Marine Science and Engineering Guangdong Laboratory (Zhuhai), Zhuhai 519082, China; 8 Department of Mechanical and Industrial Engineering, Toronto Metropolitan University (Formerly Ryerson University), Toronto, ON M5B 2K3, Canada

**Keywords:** additive manufacturing, three-dimensional (3D) printing, selective laser melting, electron beam melting, isothermal forging, Ti-6Al-4V alloy, corrosion resistance

## Abstract

3D printing (or more formally called additive manufacturing) has the potential to revolutionize the way objects are manufactured, ranging from critical applications such as aerospace components to medical devices, making the materials stronger, lighter and more durable than those manufactured via conventional methods. While the mechanical properties of Ti-6Al-4V parts manufactured with two major 3D printing techniques: selective laser melting (SLM) and electron beam melting (EBM), have been reported, it is unknown if the corrosion resistance of the 3D-printed parts is comparable to that of the alloy made with isothermal forging (ISF). The aim of this study was to identify the corrosion resistance and mechanisms of Ti-6Al-4V alloy manufactured by SLM, EBM and ISF via electrochemical corrosion tests in 3.5% NaCl solution, focusing on the effect of microstructures. It was observed that the equiaxed α + β microstructure in the ISF-manufactured Ti-6Al-4V alloy had a superior corrosion resistance to the acicular martensitic α′ + β and lamellar α + β microstructures of the 3D-printed samples via SLM and EBM, respectively. This was mainly due to the fact that (1) a higher amount of β phase was present in the ISF-manufactured sample, and (2) the fraction of phase interfaces was lower in the equiaxed α + β microstructure than in the acicular α′ + β and lamellar α + β microstructures, leading to fewer microgalvanic cells. The lower corrosion resistance of SLM-manufactured sample was also related to the higher strain energy and lower electrochemical potential induced by the presence of martensitic twins, resulting in faster anodic dissolution and higher corrosion rate.

## 1. Introduction

Titanium alloys have been extensively used in the aerospace and biomedical sectors as well as in the marine environment due to their excellent mechanical properties and superior corrosion resistance [1,2,3,4]. Ti-6Al-4V alloy is the most widely used titanium alloy with a typical alpha-beta duplex microstructure, and is also the most common one of all titanium alloys [5], where the electrochemical behavior has been extensively investigated. For example, the corrosion performance was studied by correlating the microstructure and chemical composition at grain boundaries [6,7,8]. In addition, fabrication processes and heat treatment procedures can also have a significant impact on its corrosion behavior [9,10,11,12].

Compared with the isothermal forging (ISF) technology used for titanium alloy processing [5,13], the additive manufacturing (AM) technology, also known as three-dimensional (3D) printing, for titanium alloys has many prominent features, particularly with short manufacturing cycles [14,15,16,17]. The high utilization of materials in the manufacturing of complex structural parts enables its high integral repair and weight reduction [18,19]. Indeed, 3D printing has recently taken the world by storm, since it is poised to revolutionize the way objects are manufactured, ranging from critical applications such as aerospace components to medical devices. It opens up new worlds to research in the area of materials and manufacturing, and has even become the focus of media and public attention in recent years [20,21,22,23,24,25,26,27]. The AM technique can be used for light metal alloys to fabricate lightweight structural parts in the aerospace and automotive industries. It can also be used to manufacture porous components to match the human skeletons [28,29,30].

The 3D model of the material is first designed by computer-aided design (CAD) software. Then, according to the thickness of the processing slice and the 2D shape data of each layer, the component is deposited, melted and solidified layer by layer, to eventually achieve the required part [31]. Currently, the AM techniques are mainly divided into two types: selective laser melting (SLM) and electron beam melting (EBM), according to different processing heat sources. The SLM-AM technique involves the use of fiber laser as the heating source. Since the molten layer is continuously deposited onto a cold substrate in SLM-AM, it generally has a high cooling rate and a high thermal gradient in the building direction, which leads to the occurrence of non-equilibrium martensitic phases [32]. In contrast, EBM-AM Ti-6Al-4V alloy technology uses electron beam to heat and melt powders on the substrate. Firstly, the powders in the area to be melted are preheated and sintered together to reduce the temperature gradient, which effectively avoids the generation of large internal residual stress [33]. Then, the Ti-6Al-4V powder layer undergoes rapid melting, followed by solidification in a chamber temperature maintaining in a range of 560–740 °C. Tan et al. [34] reported α′ martensitic transformation and α/β interface evolution in varied printing thicknesses of EBM-printed Ti-6Al-4V alloy block samples.

Unlike conventional manufacturing methods, AM is prone to be affected by several factors, such as power input, scan rate, scan spacing and deposition direction, which could influence the final microstructure and the resulting properties [35,36,37,38,39]. In addition, AM is essentially a rapid solidification process and large temperature gradient, which results in microstructures of components that are very different from those processed by casting, rolling and forging [40,41,42], and lead to unique changes in the corrosion resistance [4]. While a number of studies involved the mechanical properties of SLM- and EBM-manufactured parts [43,44,45], there is limited information about the corrosion resistance of the additive manufactured parts [4,6,46,47]. Dai et al. [4] reported that the unfavorable corrosion resistance of the SLM-produced sample is related to the considerably large amount of acicular α′ and less β-Ti phase in the microstructure compared with the grade 5 sample. Dai et al. [6] also reported that the XY-plane possessed a better corrosion resistance compared with the XZ-plane in 1 M HCl solution, in spite of a slight difference in 3.5 wt.% NaCl solution, suggesting that different planes exhibit different degrees of corrosion resistance in a harsher solution system, due to the presence of more α′ martensite and less β-Ti phase in the microstructure on the XZ-plane of the SLM-produced Ti-6Al-4V alloy. Zhang et al. [46] reviewed the AM of titanium alloys, including Ti-6Al-4V and Ti-24Nb-4Zr-8Sn, with a focus on manufacturing using electron beam melting (EBM) and the resultant microstructures and service properties. However, there is a lack of research on the relative corrosion resistance of titanium alloys produced via SLM, EBM and conventional isothermal forging. 

In the present study, the corrosion behavior of a Ti-6Al-4V alloy fabricated by two AM technologies and the conventional ISF method is evaluated via electrochemical techniques, with particular attention to the effect of microstructures.

## 2. Materials and Methods

**Figure 1:**
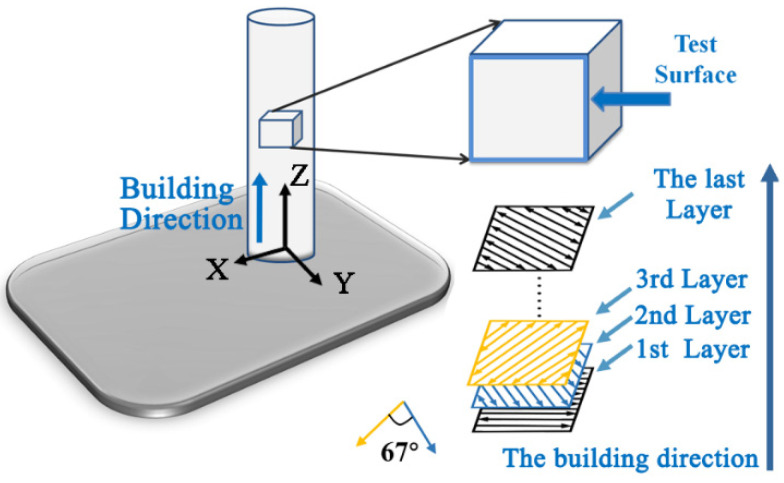
Schematic illustration of the SLM- and EBM-manufactured Ti-6Al-4V specimens built in the vertical direction.

The AM Ti-6Al-4V samples were fabricated by EBM (Arcam Q20, Gothenburg, Sweden), (pre-alloyed powder size: 45–105 μm) and SLM (Farsoon High-Technology Co., Ltd., FS271M, Changsha, China) (pre-alloyed powder size: 15–53 μm) AM techniques, respectively. All of the bars were prepared by layered slicing according to the CAD model, followed by melting, cooling and solidification layer by layer. The SLM samples were manufactured using a laser power of 340 W at a scanning rate of 1100 mm/s and a preheating temperature of 120 °C of the substrate. The SLM scan line offset and the powder layer thickness were set to be 0.11 mm and 0.06 mm, respectively. The EBM process was conducted at a vacuum of 5 × 10^−3^ mbar being charged with helium gas in the building chamber. The entire process started by preheating the powders to 500 °C with a preheating average current of 28 mA. For each layer, a focus current of 45 mA and a speed function setting of 32 were selected. The EBM samples were produced with a scan line offset of 0.22 mm, and a powder layer thickness of 0.09 mm. All of the bars were manufactured in the same direction perpendicular to the substrate (Figure 1). One step ISF Ti-6Al-4V samples were produced by our homemade isothermal forging equipment. Samples with a dimension of 10 × 10 × 10 mm were EDM cut and prepared for the microstructural observations and electrochemical corrosion experiments. The observational direction of ISF Ti-6Al-4V sample was parallel to the one-step ISF direction.

The chemical composition of the Ti-6Al-4V samples prepared by SLM, EBM and ISF is shown in Table 1. All of the samples were ground on the SiC sandpaper from grit #240 to #2000, and then polished with diamond paste down to 1 μm. The electrolyte adopted was sodium chloride solution with a mass fraction of 3.5%.

The microstructure of these samples was characterized using optical microscopy (OM, Zeiss Axio Observer.5m), scanning electron microscopy (SEM, FEI Quanta 250) equipped with energy dispersive spectroscopy (EDS), X-ray diffraction (XRD, LabX XRD-6000) and transmission electron microscopy (TEM, JEOL JEM-2100F). The mechanically polished samples were electro-polished to get rid of the deformed layer for the OM and SEM characterization. The electrolyte is Kroll’s reagent which consists of 3 mL HF, 5 mL HNO_3_ and 92 mL H_2_O. The TEM foil samples were electro-polished in a twin-jet setup at 30 V and −30 °C using the electrolyte containing 6 vol.% perchloric acid, 34 vol.% n-butanol and 60 vol.% carbinol. Samples for XRD analysis were also polished and ultrasonically cleaned before the measurements.

The corrosion performance of the samples was evaluated via an electrochemical workstation with a Galvanostat (Gamry Reference 600). Prior to the electrochemical tests, the pre-ground samples were polished to mirror finish using diamond suspension with a particle size of 1 μm. The polished samples were ultrasonically cleaned in acetone, alcohol and distilled water for ten minutes, respectively, then dried for electrochemical measurements. The electrochemical experiments were conducted in a three-electrode cell with a platinum counter electrode and a saturated calomel electrode (SCE) acted as the reference electrode at room temperature open to air. The area of the working electrode in contact with the electrolyte is 0.2827 cm^2^. Prior to any electrochemical measurements, all samples were immersed for a sufficiently long time until the open circuit potential (OCP) became nearly stable. Then potentiodynamic polarization curves were measured at a rate of 0.1667 mV/s from −500 to 1500 mV versus OCP. Electrochemical impedance spectroscopy (EIS) experiments were carried out at OCP from 10^−2^ to 10^5^ Hz with an AC amplitude of 5 mV. The results of EIS were determined using ZsimpWin 3.30 software to fit to the corresponding equivalent circuit. The electrochemical measurements were repeated three times to ensure the reproducibility of the results.

**Figure 2:**
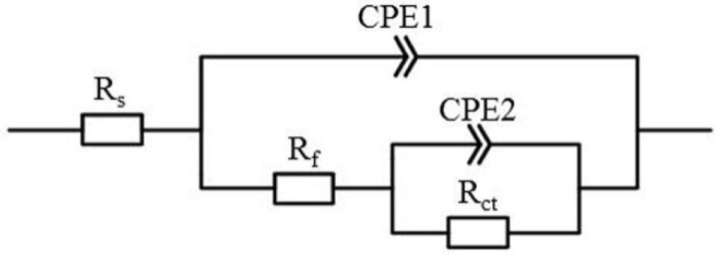
Equivalent circuits used to fit the conduction curve in the impedance spectrum analysis.

The obtained experimental EIS data were fitted using a transfer function developed for the electrical component combination in the circuit described in Figure 2. It consists of the following elements: R_s_ (electrolyte solution resistance), R_ct_ (charge transfer resistance), R_f_ (film resistance), CPE_1_ and CPE_2_ (double layer capacitance). R_f_ reflects the resistance value of the film formed on the alloy surface, while R_ct_ describes the resistance of the electrode process. Then the polarization resistance (R_p_) is the sum of the charge transfer resistance (R_ct_) and the film resistance (R_f_), i.e., R_p_ = R_ct_ + R_f_ [10]. The passivity of the samples can be evaluated via the polarization resistance: a high R_p_ indicates a higher corrosion resistance.

## 3. Results

### 3.1. Microstructure and Phase Analysis

#### 3.1.1. Metallographic Observations

**Figure 3:**
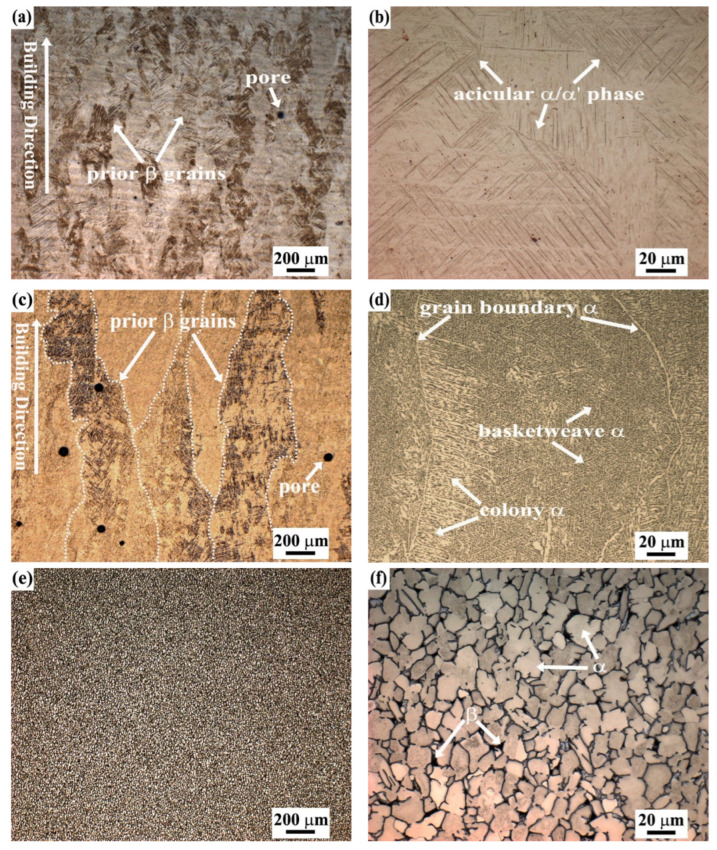
Optical images showing the microstructure of the Ti-6Al-4V alloy samples made with (**a**,**b**) SLM, (**c**,**d**) EBM and (**e**,**f**) ISF.

The typical microstructures of three Ti-6Al-4V alloy samples made with two AM techniques (SLM and EBM) and ISF are presented in Figure 3. All of them have an α + β microstructure. The microstructure of the SLM-Ti-6Al-4V alloy (Figure 3a,b) consists of a fully fine acicular α′ martensite due to the specific manufacturing process involving rapid colling [48]. It can also be observed that the macrostructure of SLM sample contains prior β columnar grains, which develop epitaxially along the building direction as marked with an arrow in Figure 3a. For the EBM-Ti-6Al-4V alloy (Figure 3c,d), in the solidification process the prior β grains were also observed with a growing direction parallel to the building direction, as indicated by the arrow in Figure 3c. Within the columnar prior β grains, fine α platelets and the interfacial rod-shaped β phase are typical (α + β) structures. The α platelet herein is a typical Widmanstatten microstructure [49], including the basket shape and a colony form. It can also be seen that the α phase was formed around the boundaries of columnar prior β grains. In addition, as a result of the AM manufacturing process, some small pores were observed in both SLM and EBM samples. The microstructure of the ISF-Ti-6Al-4V alloy (Figure 3e,f) shows an (α + β) equiaxial shaped morphology, where the brighter areas represent the α phase, while the β phase is distributed in the dark areas.

#### 3.1.2. Transmission Electron Microscopy (TEM) Observations

**Figure 4:**
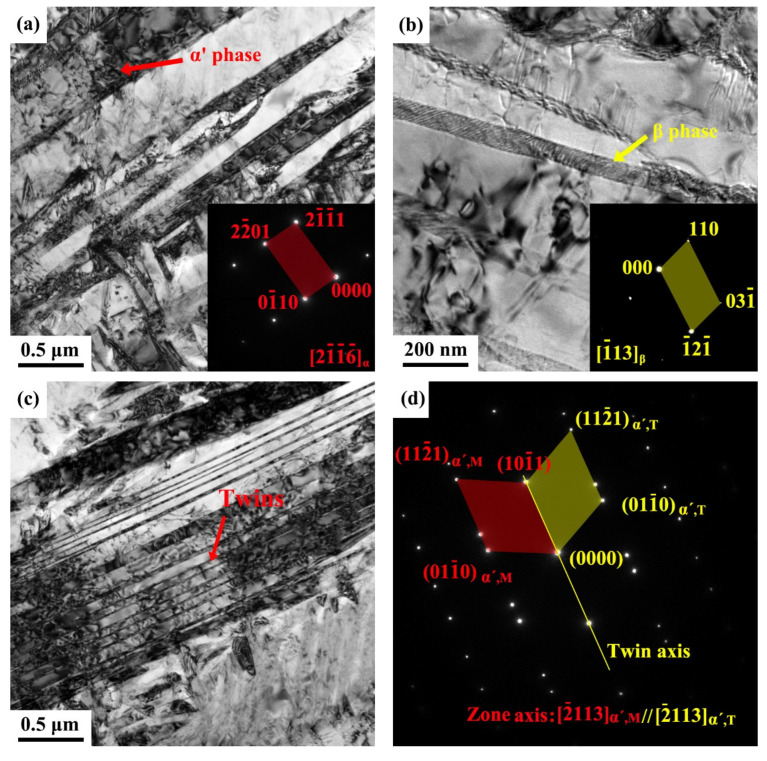

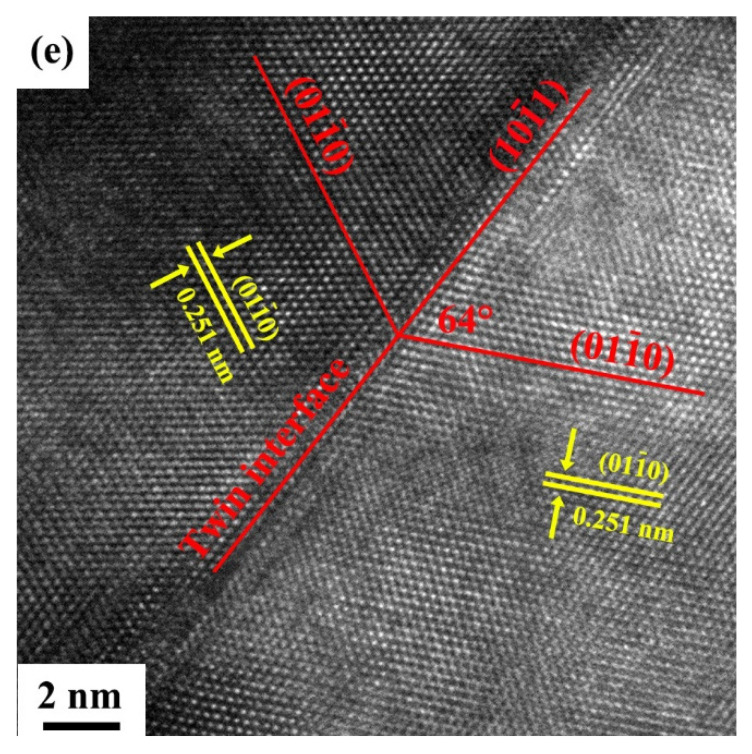
Bright-field TEM images showing the microstructure of Ti-6Al-4V alloy samples made with SLM (**a**–**c**), where the inserted SADP in (**a**,**b**) corresponds to the arrowed region on the images and (**c**) shows twins as revealed by the corresponding SADP in (**d**) which is taken from the arrowed region in (**c**), and (**e**) HRTEM image of a twin in the SLM sample.

More detailed TEM examinations were conducted with the results shown in Figure 4. The α grains in the SLM specimen are mainly acicular along with the presence of α′ martensite (Figure 4a,b). The distinctive microstructure of the SLM specimen is primarily composed of acicular α′ phase. In the region of the needle-shaped rod, TEM images (Figure 4a,b) display the α′ phase, and the β phase is positioned in-between the rods which are too small to be identified in the low-magnification OM image (Figure 3a). The phases can be revealed by the selected area diffraction patterns (SADPs), i.e., the hexagonal close-packed (HCP) α′ martensite (Figure 4a inset) and the (BCC) β-Ti (Figure 4b inset). Some dislocations in the α-Ti phase (or in-between β-Ti lamellae) can be seen to be present in the EBM specimen (Figure 4b), while a large number of twins exist in the SLM specimen (Figure 4c–e).

**Figure 5:**
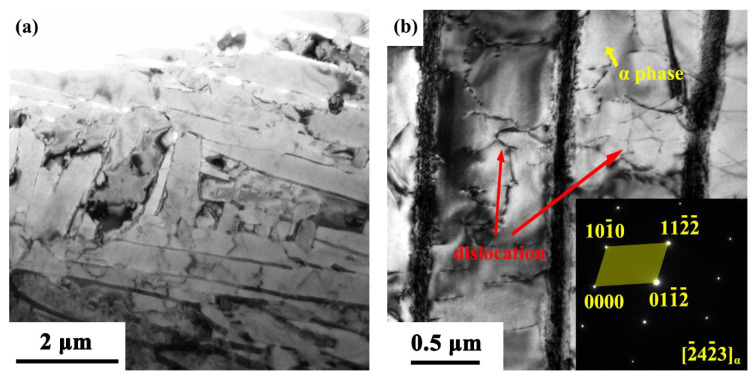

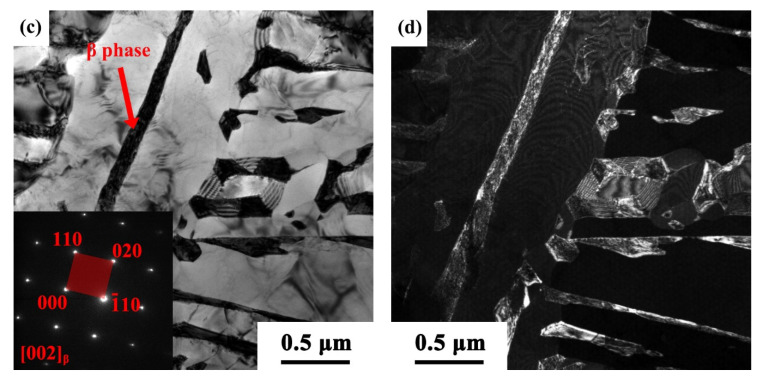
Bright-field TEM images showing the microstructure of Ti-6Al-4V alloy samples made with EBM (**a**–**c**), where the inserted SADP in (**b**) corresponds to the yellow-arrowed region, (**c**) β-phase in a bright-field TEM image and its corresponding SADP, and (**d**) dark-field TEM image of (**c**), where the β-phase becomes white.

As seen from Figure 5, the α grains in the EBM specimen are lamellar (Figure 5a,b). The typical lamellar (or massive) α + β microstructures of EBM specimens are also uncovered by the corresponding SADPs for the (HCP) α-Ti (Figure 5b inset) and β-Ti (Figure 5c inset). Some dislocations in the α-Ti phase (or in-between β-Ti lamellae) can also be seen to be present in the EBM specimen (Figure 5b–d).

#### 3.1.3. X-ray Diffraction (XRD) Analysis

**Figure 6:**
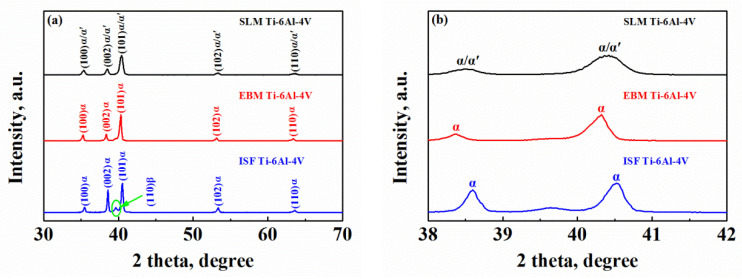
(**a**) XRD patterns of SLM- and EBM-manufactured Ti-6Al-4V and ISF alloy samples, and (**b**) magnified details of the XRD patterns in (**a**) with diffraction angles ranging from 38 to 42°.

Figure 6 shows the XRD patterns for the SLM- and EBM-manufactured Ti-6Al-4V alloys as well as the ISF alloy. As seen from Figure 6, the main phases of the three specimens are the same, consisting of α phase and a small amount of β phase. The peak intensity for the β phase in the ISF sample is higher than that of the AM-manufactured samples which is indicated in Figure 6a by the green circle, suggesting a relatively high volume fraction of β phase, while there is almost no clue for the β phase in the SLM counterpart on the XRD pattern, which revealed a mixture of primary α and martensitic α′ phase. The lattice parameters of the supersaturated martensitic α′ phase are slightly different from those of the α phase, resulting in peak broadening of all the α reflections in the SLM-manufactured Ti-6Al-4V alloy (Figure 6b). This could be judged by a full width at half maximum (FWHM) higher than 0.2° in 2θ [13,50]. In these patterns, the FWHM of α/α′ reflections rises nearly up to 0.8° in 2θ as a result of martensitic transformation.

### 3.2. Electrochemical Analysis

#### 3.2.1. Potentiodynamic Polarization

**Figure 7:**
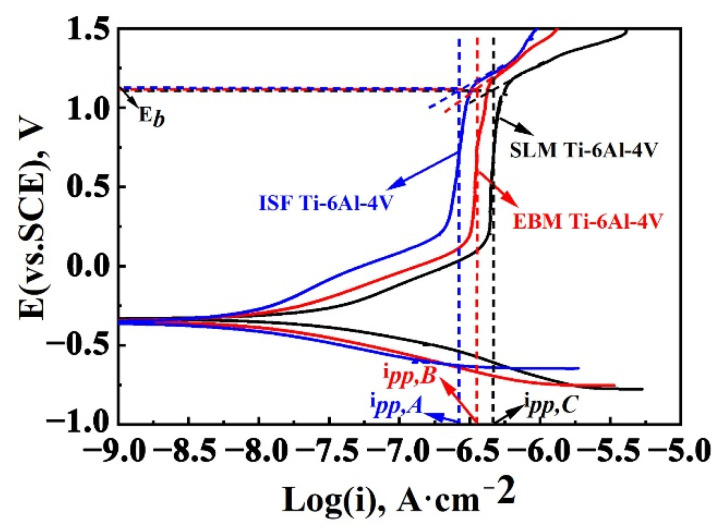
Potentiodynamic curves for the SLM- and EBM-manufactured Ti-6Al-4V samples and ISF alloy sample in a 3.5 wt.% NaCl solution.

Figure 7 shows the potentiodynamic polarization curves for the SLM- and EBM-manufactured Ti-6Al-4V alloy samples and ISF alloy sample in the 3.5 wt.% NaCl solution. Before the polarization tests, a sufficiently long time was maintained to achieve a relatively stable open circuit potential (OCP). All the samples in the NaCl solution were spontaneously passivated with a wide passivation region and a low passivation current density, indicating that all three samples have fairly good corrosion resistance. As can be seen, each polarization curve consists of both cathodic and anodic branches, and there are some differences among the three anodic polarization curves. This suggests that the oxidation reaction occurred on their surface is different. As shown in Figure 7, the transpassive potential E_b_ values, which represent a critical potential for the passive film to dissolve and break down, are nearly the same for all three types of Ti-6Al-4V alloy samples. This reveals that the passive film formed on the alloy surface is similarly stable. However, the passivation current density i_pp_ showed obvious differences. The passivation current density obtained was the smallest for the ISF alloy (i_pp,A_ = 0.20 μA/cm^2^), and largest for the SLM-manufactured Ti-6Al-4V alloy (i_pp,C_ = 0.53 μA/cm^2^), while the passivation current density for the EBM-manufactured Ti-6Al-4V alloy lay in-between these values, i.e., i_pp,B_ = 0.42 μA/cm^2^. In general, a lower passivation current density reflects a more sensitive surface to passivation in a given solution and a lower corrosion rate of the alloy [8]. Thus, it can be concluded that the ISF Ti-6Al-4V alloy exhibits the best corrosion resistance, while the corrosion resistance of the SLM- and EBM-manufactured Ti-6Al-4V alloy is lower in the 3.5 wt.% NaCl solution.

#### 3.2.2. Electrochemical Impedance Spectroscopy (EIS)

**Figure 8:**
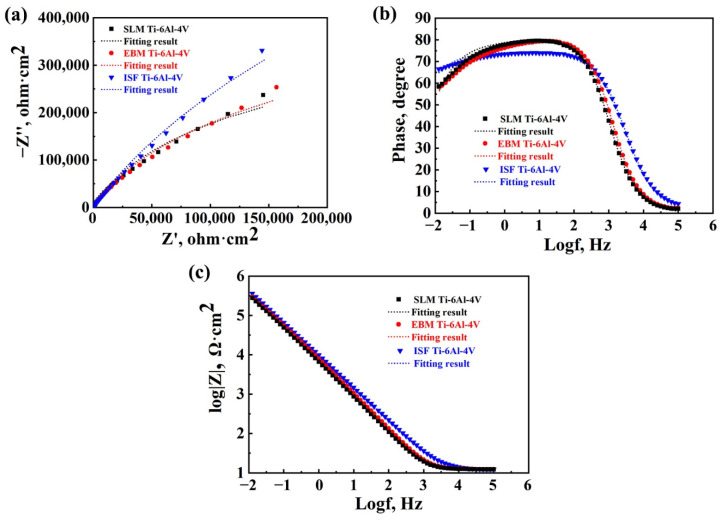
EIS results in the form of (**a**) a Nyquist plot, and (**b**,**c**) Bode plots for the SLM- and EBM-manufactured Ti-6Al-4V alloy samples and ISF alloy sample in a 3.5 wt.% NaCl solution.

The EIS measurements are shown in the form of Nyquist and Bode plots in Figure 8a–c. The passivity of the samples can be evaluated via the polarization resistance: a high R_p_ indicates a higher corrosion resistance. Figure 8a shows the AC impedance of Nyquist curves for the three samples. The capacitive arc radius of the curvature of the SLM-manufactured sample is the smallest, which suggests that the SLM-manufactured specimen has the lowest charge transfer resistance [51] and thus an inferior corrosion resistance under the test potential (a potential amplitude of 5 mV). Figure 8b,c refers to the Bode plots for the SLM- and EBM-manufactured Ti-6Al-4V alloy samples and ISF alloy sample in a 3.5 wt.% NaCl solution.

Table 2 lists the fitting results of the EIS measurements. It is clear that the R_p_ (= R_ct_ + R_f_) value is the maximum for the ISF alloy, and the minimum for the SLM-manufactured Ti-6Al-4V alloy (with more detailed analysis to be given later). This again suggests that the ISF alloy has the highest corrosion resistance, while the SLM-manufactured alloy has the lowest corrosion resistance among the three types of samples in the 3.5% NaCl solution.

Furthermore, the results of the electrical circuit fitting along with the chi-square (χ^2^) values were obtained and tabulated in Table 2, where the χ^2^ reflected the quality of fitting results. A low chi-square value indicates a good fitting quality between the experimental and the simulated data. The n_1_ values indicating a capacitive behavior of the passive film formed on the surface are 0.92, 0.98 and 0.99, for the ISF, EBM- and SLM-manufactured samples. The electrolyte solution resistance (R_s_) values are fairly similar as well for three samples in the NaCl solution considering the fitting errors. Film resistance (R_f_) reflects the resistance of the film formed on the alloy surface during passivation. There are some differences in the R_f_ values among the three samples tested in the NaCl solution, i.e., 48.3 kΩ·cm^2^, 18.8 kΩ·cm^2^ and 14.0 kΩ·cm^2^ for the ISF, EBM- and SLM-manufactured samples. This corresponds well to the current density in the passive region in Figure 7. In general, charge transfer resistance (R_ct_) is related to the rate of dissolution reaction of metals and it is the resistance of charge transfer through double layers [7]. The R_ct_ value for the SLM-manufactured Ti-6Al-4V is also much (nearly three times) smaller than that for the ISF Ti-6Al-4V. As a result, the polarization resistance (R_p_ = R_ct_ + R_f_) is highest for the ISF alloy, and lowest for the SLM-manufactured alloy. The corrosion resistance of the EBM-manufactured sample lies in-between those of the ISF and SLM-manufactured samples. This is also corroborated by the Bode impedance curves in Figure 8b,c. These findings agree well with the previous results obtained in the potentiodynamic polarization tests, as shown in Figure 7.

**Figure 9:**
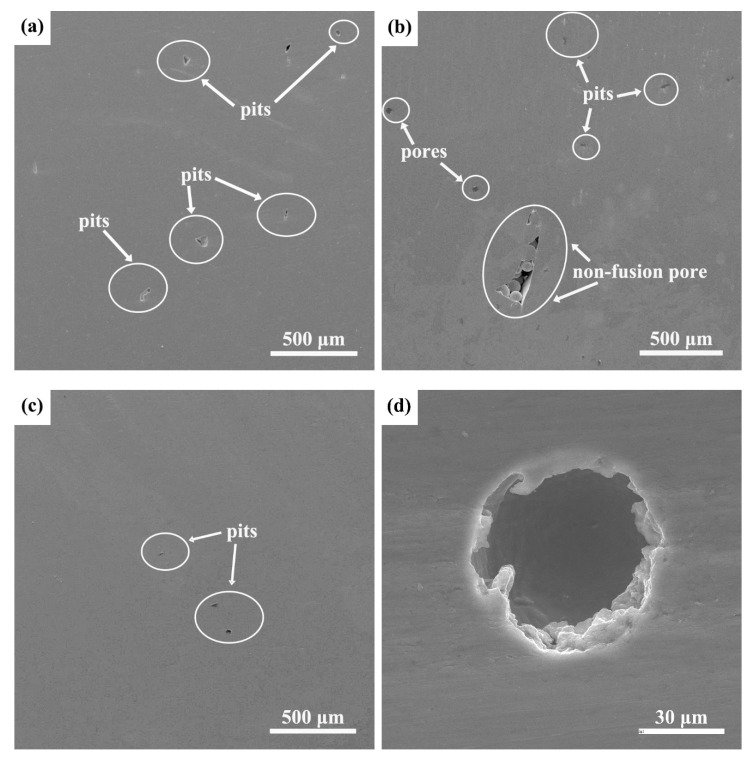
SEM images of the surface of (**a**) SLM- and (**b**) EBM-manufactured Ti-6Al-4V alloy samples, and (**c**) ISF alloy after the potentiodynamic polarization in a 3.5 wt.% NaCl solution, and (**d**) a magnified view of a hollow hole from (**b**).

The secondary electron SEM images of the ISF, EBM- and SLM-manufactured Ti-6Al-4V samples after electrochemical measurements are shown in Figure 9. It is seen that some pores (or hollow holes) were present on the surface of the EBM- and SLM-manufactured Ti-6Al-4V samples after the electrochemical corrosion tests, with a magnified image shown in Figure 9d. The corrosion pits in the three tested T-6Al-4V alloy samples increased in the following sequence: ISF alloy, EBM- and SLM-manufactured Ti-6Al-4V alloy. This suggests that the passive film formed on the SLM-manufactured alloy exhibits the worst stability and the passive film formed on the ISF Ti-6Al-4V alloy is most stable against the electrochemical corrosion in the 3.5 wt.% NaCl solution, which is in good agreement with the R_p_ values for the ISF alloy, EBM- and SLM-manufactured Ti-6Al-4V alloy in the EIS tests.

## 4. Discussion

In terms of the corrosion resistance, the material defects always have a significant effect, such as phase boundaries, grain boundaries and twin boundaries that are observed above. The XRD results (Figure 6) and metallographic observations (Figure 3) demonstrate that the ISF sample and the AM-manufactured alloy samples have different microstructures. The morphology and volume fraction of the α, β and α′ phases are different, which subsequently lead to different corrosion-resistant capacities of the three types of Ti-6Al-4V samples [4]. The relatively weak corrosion resistance in the SLM-manufactured alloy might be affected by a high density of thin needle-shaped acicular microstructures, compared with the EBM-manufactured and ISF samples containing the lamellar and equiaxed microstructures, respectively, as shown in Figure 3. Similar results have also been reported in [52,53]. The thermodynamic stability of phase boundaries between adjacent α and β phases is different. The chemical potential gradient is thus formed in the two-phase microstructure due to different compositions in the two phases. In the needle-shaped martensitic microstructure, there is a comparatively high volume fraction of interphase boundaries. Therefore, more microgalvanic cells are susceptible to formation, which promotes the occurrence of corrosion, as observed in the SLM-manufactured Ti-6Al-4V alloy. In contrast, the ISF alloy possesses an equiaxed microstructure where β phase randomly surrounds the α phase. The density of the microgalvanic cells is relatively low due to the low volume fraction of the interphase boundaries. Consequently, its corrosion resistance is expected to be higher than that of the AM-manufactured Ti-6Al-4V alloy samples.

On the other hand, in the Ti-6Al-4V alloy the β phase is known to contain more V element which plays an important part in improving its resistance to dissolution [54], as the oxide film of Ti alloy could be more stable on the β phase than on the α phase. Referring to the XRD pattern intensities (Figure 6), the volume fraction of β phase is evidently higher in the ISF sample than in the SLM- and EBM-manufactured Ti-6Al-4V samples. This difference in the volume fraction of β phase could also lead to some differences in the electrochemical corrosion behavior. As reported by other researchers [51], the higher fraction of β phase, which showed a network-like distribution along the α grain boundaries, could prevent further corrosion of the α phase matrix. Considering the different volume fractions of β phase in the three current types of samples, it is reasonable to conclude that the ISF Ti-6Al-4V alloy possesses the best corrosion resistance, while the corrosion resistance of the EBM- and SLM-manufactured Ti-6Al-4V alloy is relatively lower.

Given that the corrosion resistance between the SLM- and EBM-manufactured Ti-6Al-4V alloy samples is somewhat different, the internal defects could also be a critical factor. Electrochemical corrosion performance has relevance with the high density twins and other defects [55], simply because of the presence of high strain energy which lowers the electrochemical potential in these regions. This effect can speed up the anodic dissolution and consequently increase the corrosion rate. From the TEM observations, SLM-manufactured sample shows a large number of martensitic twins (Figure 4c–e), which would weaken its corrosion resistance, while twinning in the EBM-manufactured sample can hardly be observed. From this point of view, the slightly weaker corrosion resistance in the SLM-manufactured Ti-6Al-4V alloy in comparison with the EBM-manufactured alloy could also be understood.

## 5. Conclusions

In the present study, the corrosion behavior of Ti-6Al-4V alloy manufactured via two AM technologies (SLM and EBM) and conventional ISF method was evaluated via electrochemical corrosion tests in a 3.5% NaCl solution, focusing on the influence of microstructures including the content of β phase and defects. The following conclusions could be drawn:

1. The three fabrication methods led to different microstructures although all contained α + β phases to a varying extent. The SLM-manufactured alloy consisted mainly of fine acicular α’ martensite with a large number of twins and some prior β columnar grains, while the EBM-manufactured alloy was composed of columnar α + β structures with rod-shaped prior β grains in the building direction and fine α platelets. The microstructure of the ISF alloy exhibited a typical duplex α + β equiaxial shaped morphology. Furthermore, some small pores were observed in both SLM- and EBM-manufactured samples.
2. The equiaxed α + β microstructure of ISF sample demonstrated a better corrosion resistance than the acicular martensitic α′ + β and lamellar α + β microstructures of AM samples manufactured via SLM and EBM, respectively. This was attributed to the special benefit of the equiaxed α grains along with the randomly distributed β phase, where the fraction of phase interface was lower than that of the lamellar α + β microstructure of the EBM-manufactured alloy, leading to fewer microgalvanic cells. Additionally, the higher amount of β phase present in the ISF sample can also improve the corrosion resistance.

## Figures and Tables

**Table 1:** Chemical composition (wt.%) of SLM, EBM and ISF Ti-6Al-4V samples.

Ti-6Al-4V	Ti	Al	V	Fe	C	H	O	N
SLM	Bal.	5.50–6.75	3.50–4.50	<0.30	<0.08	<0.015	<0.20	<0.05
EBM	Bal.	6.40	4.12	0.18	0.01	0.003	0.14	0.01
ISF	Bal.	5.50–6.75	3.50–4.50	<0.30	<0.08	<0.015	<0.20	<0.05

**Table 2:** Fitting parameters of EIS for the SLM- and EBM-manufactured and ISF Ti-6Al-4V alloy samples in the 3.5 wt.% NaCl solution.

Samples	R_s_ (Ω·cm^2^)	R_f_ (kΩ·cm^2^)	CPE_1_ (F·cm^−2^)	n_1_	R_ct_ (MΩ·cm^2^)	CPE_2_ (F·cm^−2^)	*n* ^2^	χ^2^ × 10^−4^
SLM	12.3 ± 0.1	14.0 ± 0.9	59.02 ± 0.41	0.99	0.69 ± 0.04	14.87 ± 0.16	0.78	6.51
EBM	11.5 ± 0.2	18.8 ± 0.6	96.61 ± 0.93	0.98	0.89 ± 0.08	20.29 ± 0.25	0.82	6.03
ISF	10.9 ± 0.7	48.3 ± 1.1	113.91 ± 1.80	0.92	2.08 ± 0.16	41.27 ± 0.39	0.94	2.84
